# Identification of immune-related genes in diagnosing hypercholesterolemia with myocardial infarction through bioinformatics analysis

**DOI:** 10.3389/fimmu.2025.1715216

**Published:** 2026-01-06

**Authors:** Weibin Wu, Zheng Peng, Yi Yu, Zhenming Lin, Junyu Zhang, Meifang Lin, Caisheng Wu, Qiang Xie

**Affiliations:** 1 Department of Cardiology, The First Affiliated Hospital of Xiamen University, School of Medicine, Xiamen University, Xiamen, China; 2 Fujian Provincial Key Laboratory of Innovative Drug Target Research and State Key Laboratory of Cell Stress Biology, School of Pharmaceutical Sciences, Xiamen University, Xiamen, Fujian, China; 3 Department of Pathology, Zhongshan Hospital Affiliated to Xiamen University, Xiamen University, Xiamen, China

**Keywords:** hypercholesterolemia, myocardial infarction, immune infiltration, diagnosis, WGCNA

## Abstract

**Background:**

Increasing evidence suggests that familial hypercholesterolemia (FHC) exacerbates myocardial infarction (MI). This study aimed to identify possible candidate biomarkers for patients with FHC and MI.

**Methods:**

The data were obtained from Gene Expression Omnibus (GEO) database. Differentially expressed genes (DEGs) were screened using Limma, while module genes were identified through Weighted Gene Co-expression Network Analysis (WGCNA) in GSE48060. Kyoto Encyclopedia of Genes and Genomes (KEGG) and Gene Ontology (GO) enrichment analysis, protein-protein interaction (PPI) network and CIBERSORT methods were performed to explore the intersection genes. A receiver operating characteristic (ROC) curve were employed to evaluate the diagnostic effectiveness, with validation conducted using datasets GSE61144 and RT-qPCR.

**Results:**

The FHC datasets included 656 DEGs, while there were 956 DEGs and 90 module genes in MI datasets. There were 49 overlapping DEGs between FHC and MI, which were associated with immune functions. Additionally, immune infiltration analysis revealed disturbances in immune cell populations. There were 13 candiate hub genes were screen after PPI network analysis. *MCEMP1* were identified as the real hub genes after the intersection of the candiate hub genes and module genes in FHC and MI. ROC curve analysis indicated high diagnostic ability of *MCEMP1* to detect MI in GSE61144 datasets. In addition, RT-qPCR was used to detect *MCEMP1* expression in ApoE-/- mice, and the results were consistent with the bioinformatics analysis.

**Conclusion:**

*MCEMP1* were identified and provided new insights into the diagnosis and treatment of FHC with MI.

## 1 Introduction

Familial hypercholesterolemia (FHC) is a common monogenic hereditary disease characterized by significantly elevated levels of plasma low-density lipoprotein cholesterol (LDL-C), which can easily lead to atherosclerosis (1). The pathogenesis of FH is usually associated with mutations in the low-density lipoprotein receptor (LDLR), apolipoprotein B (ApoB) and proprotein convertase subtilisin/kexin type 9 (PCSK9) genes (2, 3). These gene mutations cause abnormal LDL metabolism, leading to cholesterol deposition in the vascular walls and increasing the risk of atherosclerosis and coronary heart disease. The prevalence of cardiovascular events among patients with FHC is markedly elevated compared to general population, particularly concerning myocardial infarction (MI) (4). This heightened risk is attributed to the excessive accumulation of cholesterol within the vascular walls, which predisposes the coronary arteries to narrowing or occlusion, thereby disrupting blood flow (5). Research indicates that individuals (≤ 35 years) with untreated FHC face a significantly increased likelihood of experiencing MI (6). Consequently, the early diagnosis and effective management of FHC are of paramount importance.

In recent years, there has been a notable increase in research examining the association between FHC and the occurrence of MI. Research indicates that endothelial cells in individuals with FHC exhibit increased pro-inflammatory characteristics due to extended exposure to a hypercholesterolemic milieu (7). This condition may intensify the accumulation of immune cells and the inflammatory response. Consequently, immune infiltration may significantly contribute to the early development of atherosclerosis and the elevated incidence of myocardial infarction observed in patients with FHC (8, 9). These investigations contribute to the understanding of the genetic underpinnings of FHC and its relationship with cardiovascular events, thereby establishing a foundation for clinical screening, early intervention and precision medicine. Concurrently, advancements in genetic testing technologies and bioinformatics analysis have facilitated the elucidation of the molecular mechanisms underlying FHC, paving the way for targeted molecular therapies (10, 11). However, the pathophysiology of MI in the FHC population still requires further exploration. Understanding the relationship between immune responses and FHC-related cardiovascular diseases not only helps to reveal the pathogenic mechanisms of FHC but also provides potential ideas for targeted anti-inflammatory treatments.

In this study, we acquired the FHC and MI datasets from the GEO database and utilized the Limma package to identify DEGs. Significant module genes were determined through WGCNA, followed by functional enrichment analysis. Additionally, a PPI network and CIBERSORT method were performed to explore the intersection genes. A receiver operating characteristic (ROC) curve were employed to evaluate the diagnostic effectiveness, with validation conducted using datasets GSE61144 as well as RT-qPCR in animal experiments. This research contributes to the identification of immune-related candidate biomarkers for MI in patients with FHC.

## 2 Materials and methods

### 2.1 Data collection and data processing

**Figure 1:**
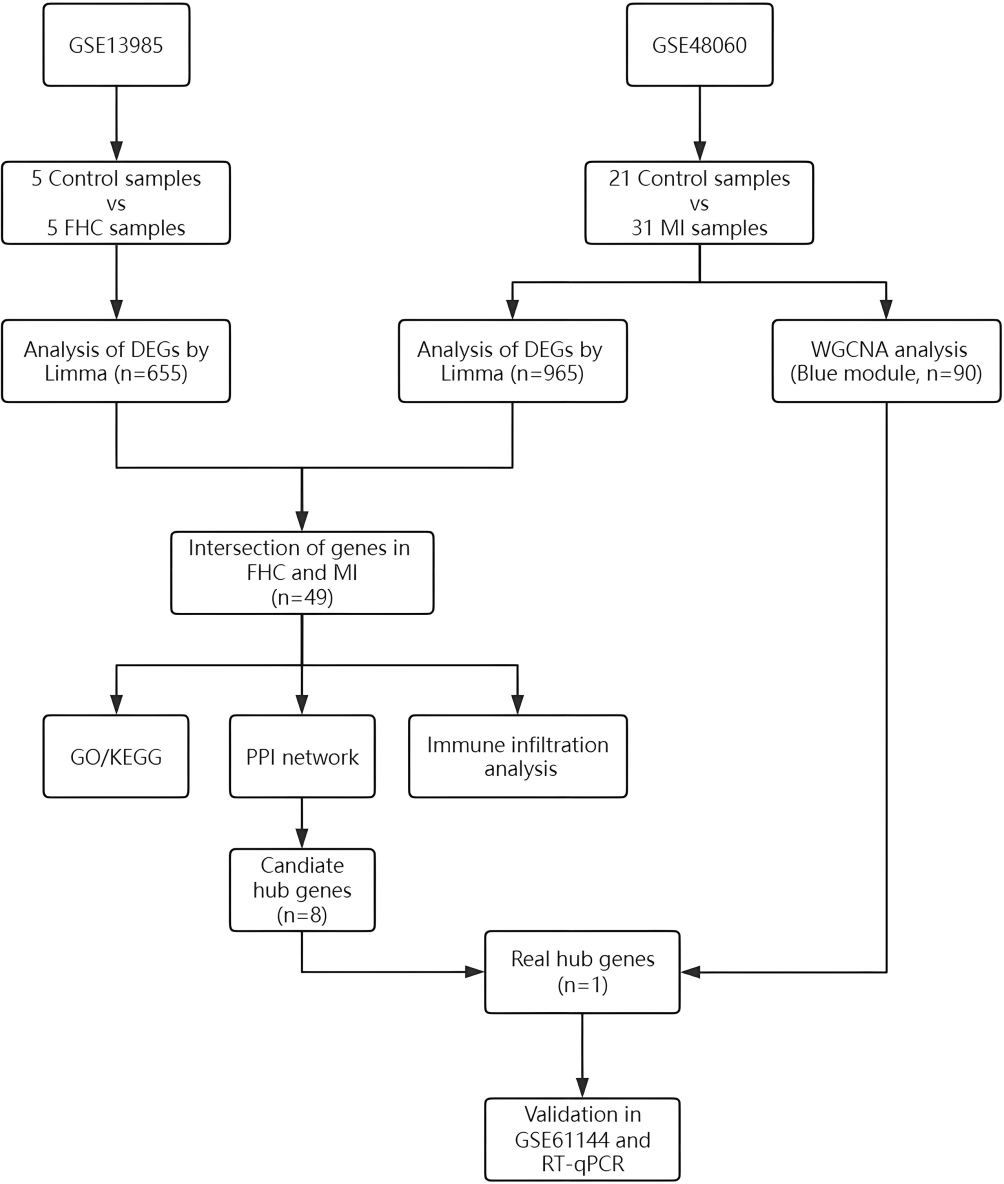
Workflow of the analysis.

We obtained three gene expression datasets from the GEO database (https://www.ncbi.nlm.nih.gov/geo/), specifically GSE13985, GSE48060 and GSE61144. The RNA expression profiles of GSE13985 and GSE48060 were both assayed on GPL570 platform, [HG-U133_Plus_2] Affymetrix Human Genome U133 Plus 2.0 Array. The RNA expression profiles of GSE61144 was assayed on GPL6106 platform, Sentrix Human-6 v2 Expression BeadChip. The GSE13985 datasets comprised 5 control samples and 5 FHC samples, the GSE48060 datasets contained 21 control samples and 31 MI samples, the GSE61144 datasets contained 10 control samples and 7 MI samples. All samples are derived from human blood. The normalization of the gene expression data was conducted utilizing the R package “optparse”. An overview of the study procedures is presented in **Figure 1**.

### 2.2 Animal experiments and sample collection

Wild-type (WT) C57BL/6 mice and ApoE^-/-^ mice on the C57BL/6 background were obtained from the Laboratory Animal Center, Xiamen University (Xiamen, China). The mice were kept in an environment with a temperature of 25 ± 2°C, humidity of 55 ± 10%, and a 12-hour light/dark cycle. Mice were divided randomly into two groups, the WT group and the ApoE^-/-^ group. All animal care and experimental procedures were approved by the Animal Ethics Committee of Xiamen University (Animal Ethics Approval No.: XMULAC20220126). All experimental procedures were conducted in strict compliance with the relevant guidelines and regulations established by the Institutional Animal Care and Use Committee of Xiamen University, and were performed in accordance with the ARRIVE guidelines for reporting animal research.

### 2.3 Differentially expressed gene screening

DEGs were identified between the FHC and control samples with a *p*-value < 0.01 and |Log_2_ fold change (FC)| > 1 in GSE13985 datasets. Similarly, DEGs were determined between the MI and control samples with a *p*-value < 0.01 and |log_2_FC| > 1 in GSE48060 datasets. The analysis was conducted using the Limma package in R software. The visualization of the DEGs was performed using the Sangerbox platform (http://vip.sangerbox.com/) (12).

### 2.4 Weighted gene correlation network analysis

In this study, WGCNA package within R software was employed to examine the relationship between genes and phenotypes through the development of a gene co-expression network. Initially, all DEGs in GSE48060 were analyzed using the WGCNA package, and the optimal soft thresholding power was established. Subsequently, a weighted co-expression network was constructed, resulting in the clustering of DEGs into several modules, each designated with distinct color labels. We then investigated the correlation between each module and MI or control groups. The module exhibiting the strongest correlation with MI was identified as a key module for subsequent enrichment analysis.

### 2.5 Function enrichment analysis

To investigate the biological roles of genes, we employed the “clusterProfile” package within R software. Initially, we conducted Gene Ontology (GO) and Kyoto Encyclopedia of Genes and Genomes (KEGG) analyses, applying a significance threshold of *p* < 0.05. Subsequently, we delineated the overlap of DEGs present in both MI and FHC samples. Following this, we conducted GO and KEGG analyses grounded in these intersecting DEGs. The resulting data were visualized using the Sangerbox platform.

### 2.6 Protein-protein interaction network and hub gene identification

The Search Tool for the Retrieval of Interacting Genes/Proteins (STRING, http://string-db.org/) was used to construct the protein-protein interaction (PPI) network of the intersection genes of MI and FHC samples. The PPI network was then visualized by Cytoscape software and then Cytohubba (13) was used to determine the candiate hub genes. Only the intersecting genes are selected for further analysis.

### 2.7 Immune infiltration analysis

To evaluate the infiltration of immune cells based on gene expression profiles, we utilized CIBERSORT (https://cibersortx.stanford.edu/), a dedicated analytical instrument. We analyzed the distribution of immune cell types within both the MI and FHC samples utilizing this platform (14). A bar plot was generated to illustrate the relative proportions of different immune cell types, while a vioplot was utilized to compare these proportions between the MI and control samples, as well as between FHC and control samples. Additionally, a heatmap created using the Sangerbox platform was employed to represent the relationships among various immunocytes (15).

### 2.8 RNA extraction and quantitative RT-PCR

According to the manufacturer’s instructions, total RNA was extracted from aorta and heart tissue using the Animal Tissue/Cell RNA Rapid Extraction Kit (Heruibio, HRQ0272, China). cDNA synthesis was carried out using HRbio™ III 1st Strand cDNA Synthesis SuperMix for qPCR (Heruibio, HRF0182, China). We utilized GAPDH as a reference for quantitative RT-PCR, employing HRbio™ qPCR SYBR Green Master Mix (Heruibio, HRF0052, China) on the ABI QuantStudio6 (Thermo Fisher Scientific, USA). The relative mRNA expression levels were calculated using the 2-ΔΔCt method. The primer pairs used for amplification were as follows: *MCEMP1*:

F: 5’-CTGCATCGTCCTCTCTGCTT-3’,
R: 5’-CTCCCGGATATTCCACACCG-3’;

GAPDH:

F: 5’-TGGAAAGCTGTGGCGTGATG-3’,
R: 5’-TACTTGGCAGGTTTCTCCAGG-3’.

### 2.9 Statistical analysis

All statistical analyses were performed in the R language (Version 4.0.3). All statistical tests were bilateral, and *p*-value < 0.05 was statistically significant. The development of the ROC curve, along with the computation of the area under the ROC curve was carried out utilizing MedCalc Statistical Software version 14.8.1 (MedCalc Software Ltd, Ostend, Belgium).

## 3 Results

### 3.1 Identifications of DEGs

**Figure 2:**
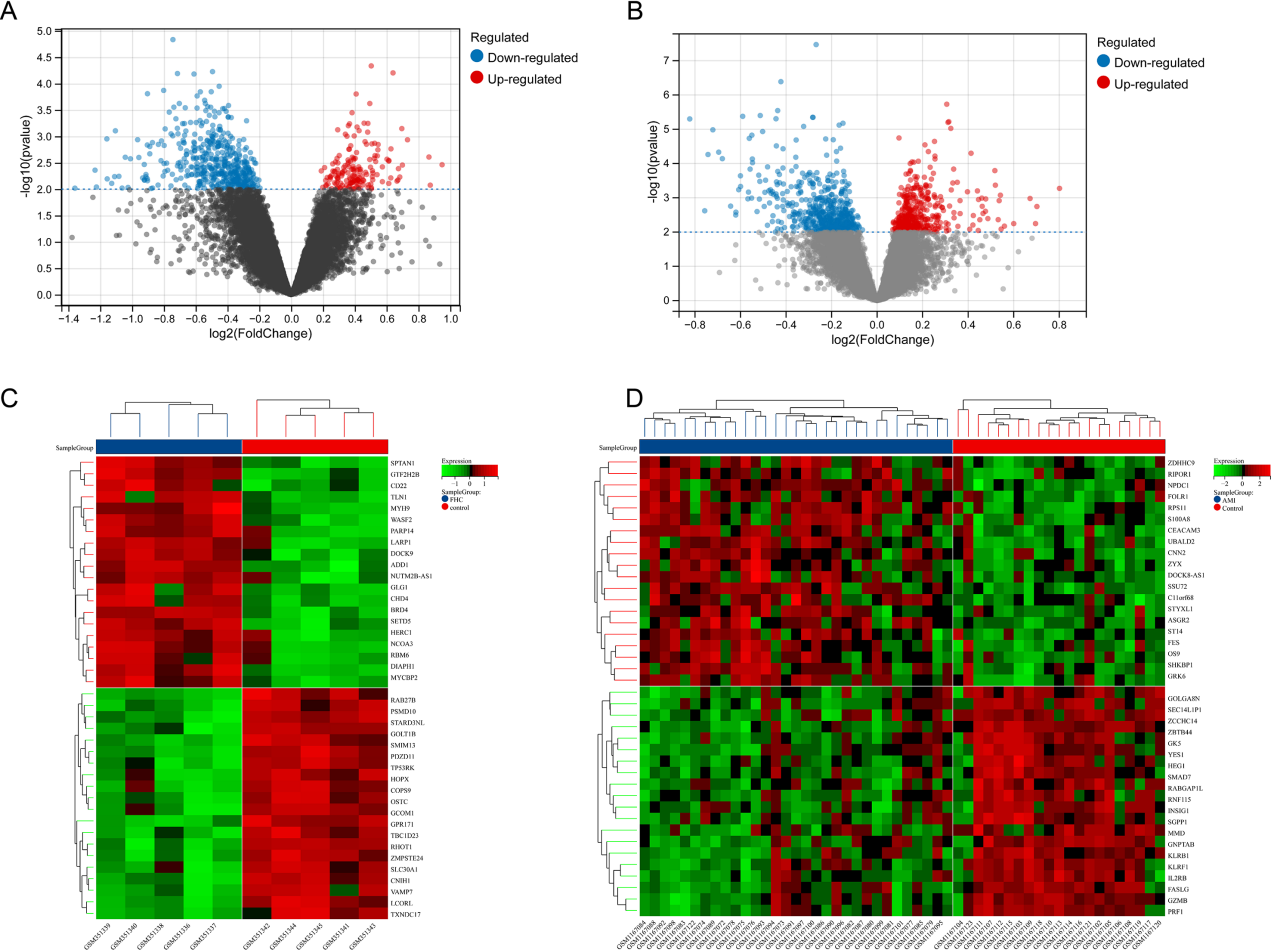
Expression profile of DEGs. **(A)** The volcano map of DEGs in GSE13985 datasets. **(B)** The volcano map of DEGs in GSE48060 datasets. **(C)** Heatmap of top 40 DEGs in GSE13985 datasets. **(D)** Heatmap of top 40 DEGs in GSE48060 datasets.

A total of 656 DEGs were identified from the GSE13985 datasets with a *p*-value < 0.01 and |log2FC| > 1. The volcano plot and heatmap presented in **Figures 2A, C**, respectively, illustrate the differential expression patterns of these FHC-related DEGs. Similarly, for the GSE48060, a total of 965 DEGs were identified using the same cutoff criteria of *p*-value < 0.01 and |log2FC| > 1. The volcano plot and heatmap in **Figures 2B, D** illustrate the differential expression profiles of these DEGs related to MI.

### 3.2 Weighted gene co-expression network analysis and critical module identification

**Figure 3:**
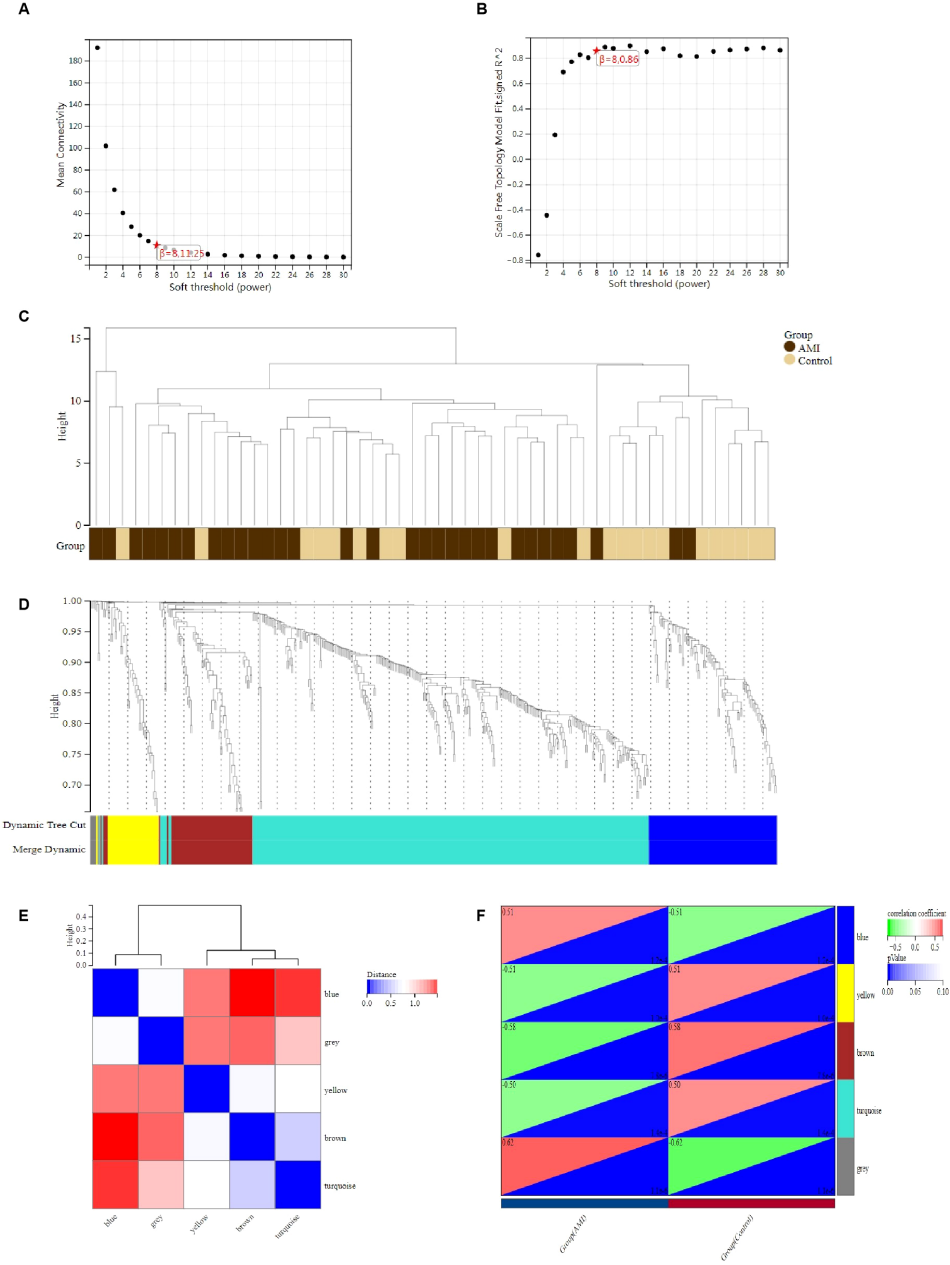
Identification of DEGs via WGCNA module genes in MI. **(A, B)** β=8 is chosen as the soft threshold based on the scale independence and average connectivity. **(C)** Clustering dendrogram of the MI and control samples. **(D)** Gene co-expression modules with different colors under the gene tree. **(E)** Heatmap of eigengene adjacency. **(F)** Heatmap of correlation between module genes and MI shows that the blue module has the highest association with MI. For each pair, the top left triangle is colored to represent the correlation coefficient; the bottom right one is colored to indicate the p-value.

We constructed a scale-free co-expression network using WGCNA to identify the most associated module in MI. “soft” threshold β of 8 was chosen based on the scale independence and average connectivity (**Figures 3A, B**). The clustering dendrogram of MI andcontrol was generated, and 5 gene co-expression modules indifferent colors were obtained with a module merge threshold of 0.25 and a minimum size of 30, as shown in **Figures 3C–E**. Clinical correlation analysis results showed that the blue module had the highest association (r = 0.51, *p*-value < 0.001) with MI in **Figure 3F**. Thus, we selected the blue module, which consisted of 90 genes, for further analysis. These results indicated that the genes in the blue module were most closely related to MI.

### 3.3 Functional enrichment analysis of FHC and MI

**Figure 4:**
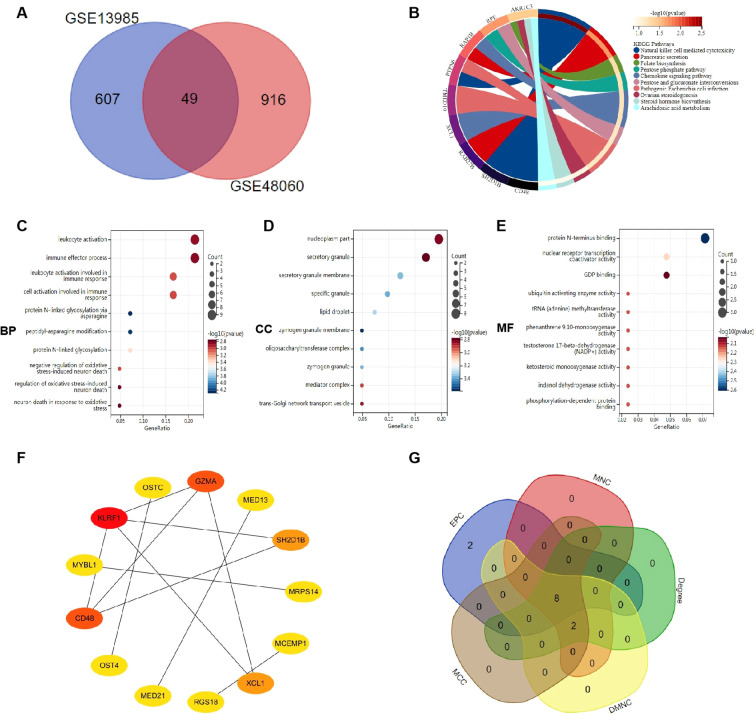
Function enrichment analysis of the intersection of genes for FHC and MI. **(A)** The intersection of DEGs via Limma genes includes 49 genes, which were shown in the Venn diagram. **(B)** KEGG analysis of the intersection of genes. Various significant pathways and associated genes are represented with different colors. **(C-E)** The GO analysis includes biological process, cellular component, and molecular function. The y-axis represents GO terms, and the x-axis represents gene ratio involved in corresponding GO terms. The size of circles represents gene numbers, and their color refers to p-value. **(F)** The PPI network demonstrates that 14 genes interact with each other. **(G)** The overlapped hub genes from different algorithms.

To validate the reliable extent of GSE13985 and GSE48060, we implemented enrichment analysis for the intersection of genes from Limma genes. A total of 49 common genes were obtained, as shown in **Figure 4A**. KEGG analysis elucidated that common genes were involved in “Natural killer cell mediatedcytotoxicity” and “Pancreatic secretion”, as shown in **Figure 4B**. The results of GO analysis revealed that common genes were enriched in biological process (BP) terms, including “leukocyte activation”, “immune effector process” and “leukocyte activation involved inimmune response”, as shown in **Figure 4C**. For cellular component (CC) ontology, the common genes are involved in “nucleoplasm part”, “secretory granule” and “secretory granule membrane”, as shown in **Figure 4D**. For molecular function (MF), the results showed that “protein N-terminus binding” was the most significant term in common genes, as shown in **Figure 4E**. The results showed that the common genes for FHC and MI were associated with immune response, which were highly related to the pathogenesis of FHC and MI.

### 3.4 Protein-protein interaction network construction

The 49 crossover genes obtained by hybridization were imported into the STRING database, the proteins interacting with them were screened, and the results obtained were imported into Cytoscape software to construct a PPI network (**Figure 4F**). The top ten hub genes obtained by five algorithms, MCC, DMNC, MNC, Degree, and EPC, in the cytohubba plug-in, are shown in **Table 1**. The overlapped hub genes among the five algorithms were verified by a Venn diagram (**Figure 4G**), including *MCEMP1*, *CD48*, *XCL1*, *SH2D1B*, *MED21*, *MED13*, *KLRF1* and *GZMA*.

**Table 1:** Top ten hub genes obtained by five algorithms of Cytohubba.

MCC	DMNC	MNC	Degree	EPC
*KLRF1*	*CD48*	*KLRF1*	*KLRF1*	*KLRF1*
*CD48*	*GZMA*	*CD48*	*CD48*	*GZMA*
*GZMA*	*SH2D1B*	*GZMA*	*GZMA*	*CD48*
*SH2D1B*	*XCL1*	*SH2D1B*	*SH2D1B*	*SH2D1B*
*XCL1*	*KLRF1*	*XCL1*	*XCL1*	*XCL1*
*MCEMP1*	*MCEMP1*	*MCEMP1*	*MCEMP1*	*OST4*
*RGS18*	*RGS18*	*RGS18*	*RGS18*	*OSTC*
*MED13*	*MED13*	*MED13*	*MED13*	*MED13*
*MED21*	*MED21*	*MED21*	*MED21*	*MED21*
*MRPS14*	*MRPS14*	*MRPS14*	*MRPS14*	*MCEMP1*

### 3.5 Evaluation of immune cell infltration and immune cell correlation analysis

**Figure 5:**
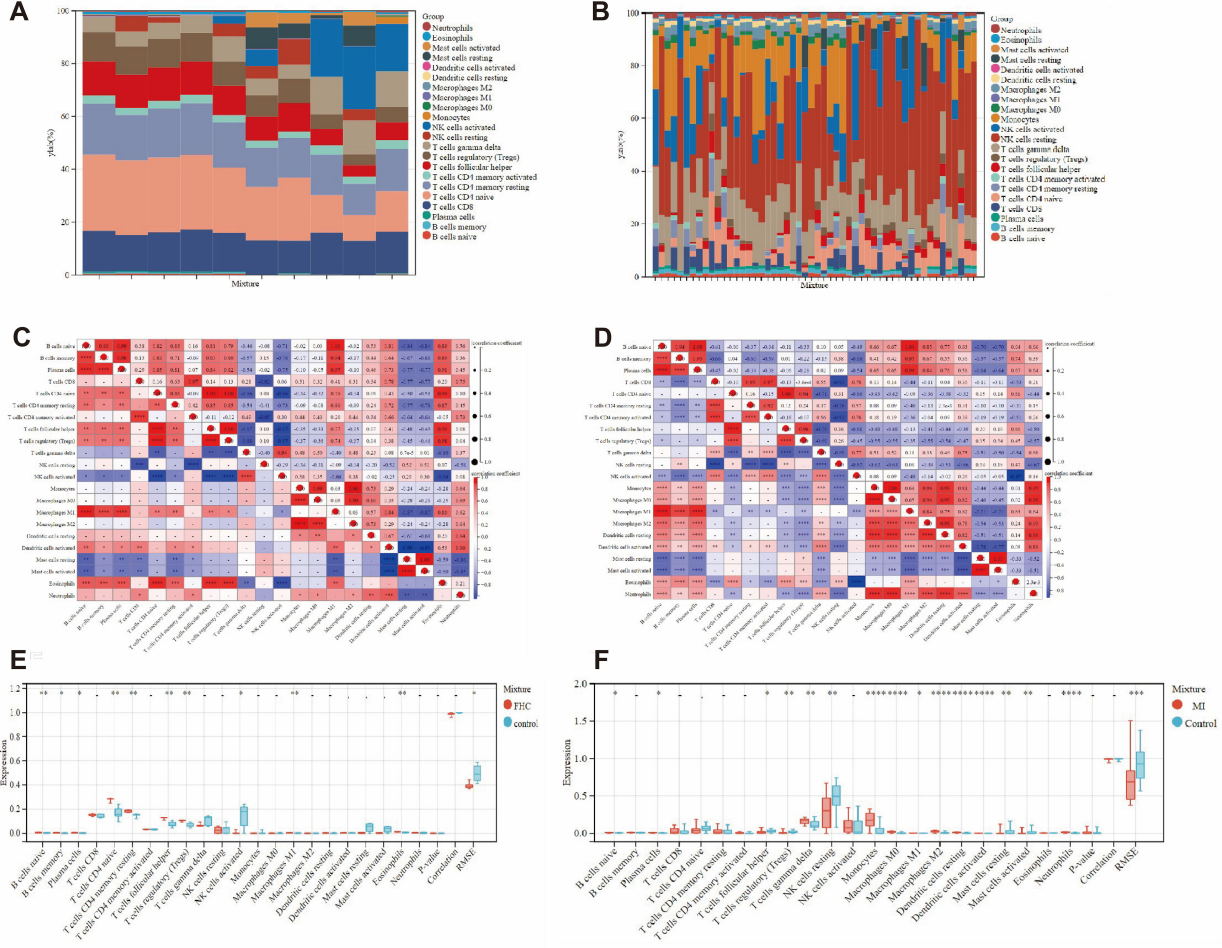
Immune infiltration analysis between FHC or MI and control. **(A)** The proportion of 22 immunocytes in all samples visualized from the bar plot in GSE13985. **(B)** The proportion of 22 immunocytes in all samples visualized from the bar plot in GSE48060. **(C)** Comparison of the proportion of 22 kinds of immunocytes between FHC and control groups shown in the vioplot. **(D)** Comparison of the proportion of 22 kinds of immunocytes between MI and control groups shown in the vioplot. **(E)** Association of 22 immunocyte-type compositions in GSE13985. **(F)** Association of 22 immunocyte-type compositions in GSE48060. *p < 0.05; **p < 0.01; ***p < 0.001, ****p < 0.0001.

Because the key diagnosis genes that were correlated with FHC and MI can regulate the pathogenesis of FHC and MI, which were mainly enriched in immunity. The immune infiltration analysis can better explore the effect of immunity in FHC and MI. For FHC or MI and the control groups, the proportion of 22 kinds of immunocytes are shown in **Figure 5A, B**. The box plot presented that compared with the control group, NK cells activated had a lower level in the FHC group, while B cells naive, B cells memory, Plasma cells, T cells CD4 naive, T cells CD4 memory resting, T cells follicular helper, T cells regulatory (Tregs), Macrophages M1 and Eosinophils had a high level, as shown in **Figure 5E**. The correlation of 22 types of immunocytes demonstrates that B cells naive were positively related to Macrophages M1 (r = 1), Dendritic cells activated were negatively related to Mast cells resting (r = -0.99), Mast cells activated were negatively related to Dendritic cells activated (r = -0.99) in FHC, and all the associations are shown in **Figure 5C**. Similarly, The box plot illustrated that, compared with the control group, T cells follicular helper levels, Tregs levels, NK cells resting levels, Mast cells resting and RMSE levels were lower in the FHC group, whereas levels of B cells naive, Plasma cells, T cells gamma delta, Monocytes, Macrophages M0, Macrophages M1, Macrophages M2, Dendritic cells resting and Neutrophils were higher, as depicted in **Figure 5F**. The analysis of 22 types of immune cells revealed a positive correlation between B cells naive and macrophages M1 (r = 1), while NK cells activated showed a negative correlation with Eosinophils (r = -0.87) in MI group. All these relationships are illustrated in **Figure 5D**. In summary, the different level of infiltration of immunocytes in FHC or MI patients may serve as a potential treatment target.

### 3.6 Identifications and validation of real hub genes

**Figure 6:**
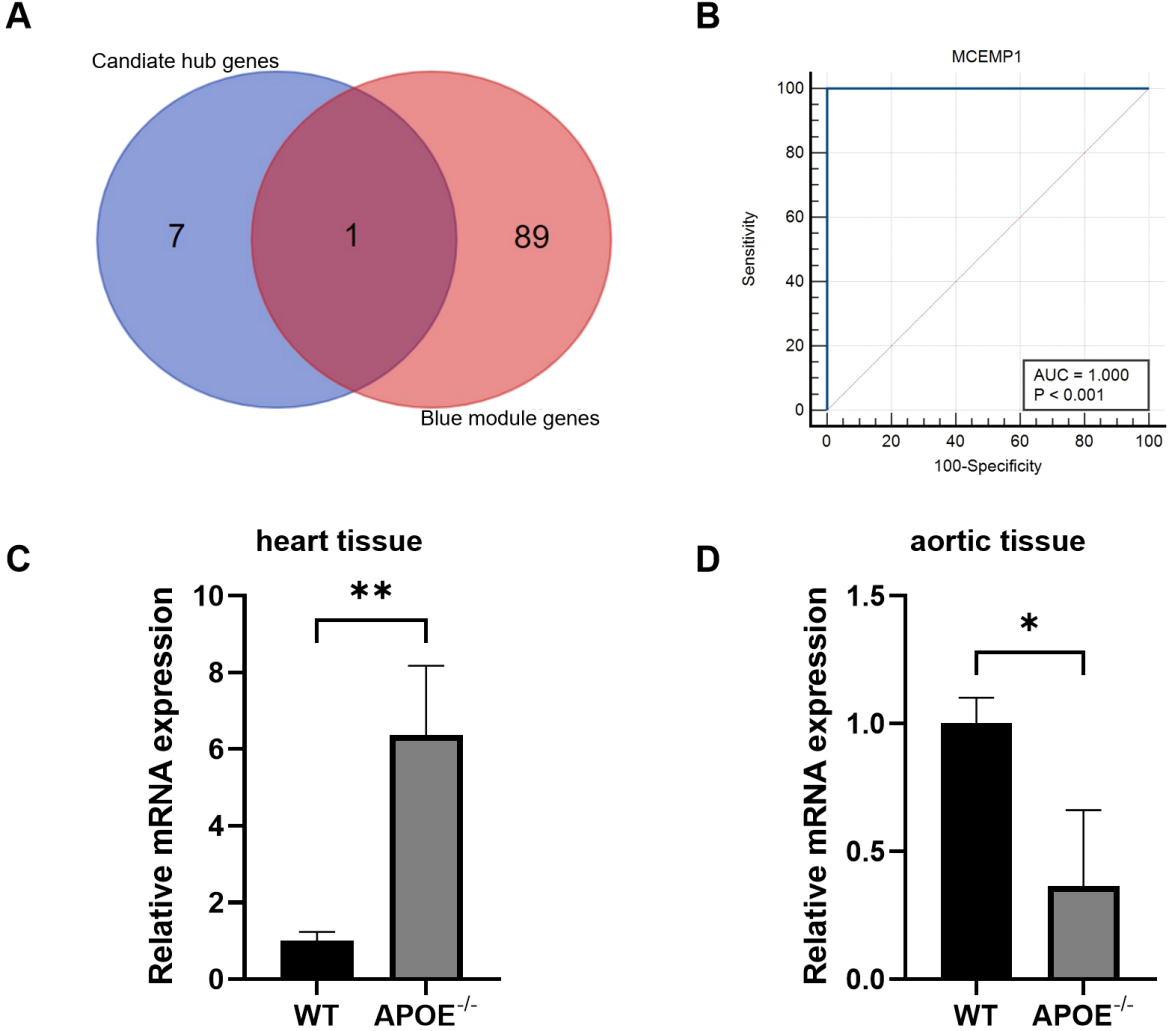
Validation of hub genes. **(A)** The overlapped genes from candiate hub genes and blue module genes. **(B)** ROC curve for *MCEMP1* in GSE61144 datasets. **(C)** Relative mRNA levels of *MCEMP1* in the heart tissues of WT and ApoE^-/-^ groups. **(D)** Relative mRNA levels of *MCEMP1* in the aortic tissues of WT and ApoE^-/-^ groups *p < 0.05; **p < 0.01.

After the intersection of candiate hub genes and blue module genes from WGCNA analysis, *MCEMP1* were identified as the real hub genes in FHC and MI (**Figure 6A**). ROC curve for *MCEMP1* in GSE61144 datasets indicated high diagnostic ability of *MCEMP1* to detect MI (**Figure 6B**). RT-qPCR analysis revealed that *MCEMP1* expression was markedly increased in the heart tissue of ApoE^-/-^ mice, while it was significantly decreased in the aorta. These findings further corroborate the reliability of the bioinformatics analysis results.

## 4 Discussion

The intricate relationship between immune infiltration and the pathogenesis in FHC patients with MI has been a focal point of recent research. Our study underscores the critical role that immune mechanisms play in exacerbating cardiovascular complications in FHC patients. By elucidating the underlying immunological pathways, we gain a deeper understanding of how these processes contribute to the heightened risk of myocardial infarction in this genetically predisposed population.

The evidence presented across various studies reveals a complex interplay between lipid metabolism, inflammation, and immune response (16–18). This multifaceted interaction not only highlights the necessity for a holistic approach in managing FHC but also opens new avenues for therapeutic interventions. Specifically, targeting immune modulation could offer a novel strategy to mitigate the cardiovascular risks associated with FHC.

Inflammation is part of the body’s innate immune response, or rather, it is the process the body uses to summon immune cells to fend off pathogens. Mast cells can release histamine and trigger other immune responses, leading to allergic inflammation in the body (19). Therefore, researchers analyzed a surface protein on mast cells, *MCEMP1*, which is crucial for eliciting severe immune responses in the body (20). The *MCEMP1* protein functions by encoding a single-pass transmembrane protein and is involved in the regulation of monocyte differentiation and immune response processes (21). Previous research has indicated that, in addition to being associated with asthma, *MCEMP1* is also related to various inflammatory lung diseases, such as idiopathic pulmonary fibrosis (IPF) (22). When the expression of *MCEMP1* is eliminated from the surface of mast cells, researchers observed a decrease in airway inflammation and lung damage levels. They also found that *MCEMP1* is associated with an increase in the number of mast cells in the body; when *MCEMP1* is expressed in mast cells, researchers can observe a higher incidence of inflammation and lung function deficits in the body (20). In this study, we systematically analyzed the differentially expressed genes in FHC and MI using bioinformatics analysis methods, and constructed a scale-free co-expression network using WGCNA to identify the modules most relevant to MI. By intersecting candidate hub genes with the blue module genes from the WGCNA analysis, we identified *MCEMP1* as a true hub gene in FHC and MI. The results of the GO and KEGG analyses revealed a significant involvement of *MCEMP1* in the pathogenesis of immune responses in FHC-MI. Furthermore, the results of immune infiltration analysis revealed disturbances in immune cell populations. ROC curve analysis and RT-qPCR results indicate that *MCEMP1* may serve as a candidate biomarker for further study for MI and FHC patients.

However, our study had certain limitations. Firstly, it only obtained two gene expression datasets from the GEO database. Unfortunately, we were unable to validate the findings using external datasets outside of the GEO database. In the future, the scale and diversity of the datasets can be further expanded to achieve more comprehensive and accurate research results. Secondly, there is still room for further exploration of the pathogenesis of FHC and MI in the study. Future research could incorporate more experimental techniques and methods, such as cell experiments and clinical study, to investigate the pathogenesis from multiple levels. Thirdly, the ApoE^-/-^ mouse model has been widely accepted for studying atherosclerosis and cardiovascular disease, despite its limitations in fully replicating the LDL-driven pathology of FHC. The rationale for this choice is based on the model’s ability to develop hypercholesterolemia and atherosclerotic lesions, which are relevant to the study of MI comorbidity. However, we agree that the model’s focus on triglyceride-rich lipoprotein clearance rather than elevated LDL is a notable limitation. Finally, regarding the results of the immune infiltration analysis, future studies could further explore the specific relationship between the differences in immune cell infiltration levels and the pathogenesis of FHC and MI, as well as how to better apply this in clinical treatment. For instance, based on the identification of the true hub gene *MCEMP1*, future research could delve deeper into its mechanism of action and how to develop more effective treatment strategies targeting this gene.

In conclusion, this study revealed the common molecular characteristics of FHC and MI through multi-omics integrative analysis, identifying key genes such as *MCEMP1* and immune-related pathways. These findings not only deepen the understanding of the mechanisms of heart diseases but also provide important clues for the development of new candidate biomarker. Future research could combine clinical sample validation and mechanism exploration to promote translational applications.

## Data Availability

The datasets presented in this study can be found in online repositories. The names of the repository/repositories and accession number(s) can be found below: https://www.ncbi.nlm.nih.gov/, GSE13985 https://www.ncbi.nlm.nih.gov/, GSE48060 https://www.ncbi.nlm.nih.gov/, GSE61144.

## References

[1] Zhao H Li Y He L Pu W Yu W Li Y . *In vivo* AAV-CRISPR/cas9-mediated gene editing ameliorates atherosclerosis in familial hypercholesterolemia. Circulation. (2020) 141:67–79. doi: 10.1161/CIRCULATIONAHA.119.042476, PMID: 31779484

[2] Raal FJ Kallend D Ray KK Turner T Koenig W Wright RS . Inclisiran for the treatment of heterozygous familial hypercholesterolemia. N Engl J Med. (2020) 382:1520–30. doi: 10.1056/NEJMoa1913805, PMID: 32197277

[3] Wiegman A Greber-Platzer S Ali S Reijman MD Brinton EA Charng MJ . Evinacumab for pediatric patients with homozygous familial hypercholesterolemia. Circulation. (2024) 149:343–53. doi: 10.1161/CIRCULATIONAHA.123.065529, PMID: 37860863 PMC10814999

[4] Santos RD Gidding SS Hegele RA Cuchel MA Barter PJ Watts GF . Defining severe familial hypercholesterolaemia and the implications for clinical management: a consensus statement from the International Atherosclerosis Society Severe Familial Hypercholesterolemia Panel. Lancet Diabetes Endocrinol. (2016) 4:850–61. doi: 10.1016/S2213-8587(16)30041-9, PMID: 27246162

[5] Yao H Farnier M Tribouillard L Chague F Brunel P Maza M . Coronary lesion complexity in patients with heterozygous familial hypercholesterolemia hospitalized for acute myocardial infarction: data from the RICO survey. Lipids Health Dis. (2021) 20:45. doi: 10.1186/s12944-021-01467-z, PMID: 33947397 PMC8094609

[6] Li S Zhang HW Guo YL Wu NQ Zhu CG Zhao X . Familial hypercholesterolemia in very young myocardial infarction. Sci Rep. (2018) 8:8861. doi: 10.1038/s41598-018-27248-w, PMID: 29892007 PMC5995844

[7] Don-Doncow N Vanherle L Matthes F Petersen SK Matuskova H Rattik S . Simvastatin therapy attenuates memory deficits that associate with brain monocyte infiltration in chronic hypercholesterolemia. NPJ Aging Mech Dis. (2021) 7:19. doi: 10.1038/s41514-021-00071-w, PMID: 34349106 PMC8338939

[8] Dong R Li J Jiang G Han N Zhang Y Shi X . Novel immune cell infiltration-related biomarkers in atherosclerosis diagnosis. PeerJ. (2023) 11:e15341. doi: 10.7717/peerj.15341, PMID: 37151293 PMC10158768

[9] Singh A Gupta A Collins BL Qamar A Monda KL Biery D . Familial hypercholesterolemia among young adults with myocardial infarction. J Am Coll Cardiol. (2019) 73:2439–50. doi: 10.1016/j.jacc.2019.02.059, PMID: 31097165

[10] Udhaya Kumar S Thirumal Kumar D Bithia R Sankar S Magesh R Sidenna M . Analysis of differentially expressed genes and molecular pathways in familial hypercholesterolemia involved in atherosclerosis: A systematic and bioinformatics approach. Front Genet. (2020) 11:734. doi: 10.3389/fgene.2020.00734, PMID: 32760426 PMC7373787

[11] Liu MM Peng J Guo YL Zhu CG Wu NQ Xu RX . SORBS2 as a molecular target for atherosclerosis in patients with familial hypercholesterolemia. J Transl Med. (2022) 20:233. doi: 10.1186/s12967-022-03381-z, PMID: 35590369 PMC9118763

[12] Chen D Xu L Xing H Shen W Song Z Li H . Sangerbox 2: Enhanced functionalities and update for a comprehensive clinical bioinformatics data analysis platform. Imeta. (2024) 3:e238. doi: 10.1002/imt2.238, PMID: 39429873 PMC11487553

[13] Chin CH Chen SH Wu HH Ho CW Ko MT Lin CY . cytoHubba: identifying hub objects and sub-networks from complex interactome. BMC Syst Biol. (2014) 8 Suppl 4:S11. doi: 10.1186/1752-0509-8-S4-S11, PMID: 25521941 PMC4290687

[14] Newman AM Liu CL Green MR Gentles AJ Feng W Xu Y . Robust enumeration of cell subsets from tissue expression profiles. Nat Methods. (2015) 12:453–7. doi: 10.1038/nmeth.3337, PMID: 25822800 PMC4739640

[15] Shen W Song Z Zhong X Huang M Shen D Gao P . Sangerbox: A comprehensive, interaction-friendly clinical bioinformatics analysis platform. Imeta. (2022) 1:e36. doi: 10.1002/imt2.36, PMID: 38868713 PMC10989974

[16] Xu Q Zhao YM He NQ Gao R Xu WX Zhuo XJ . PCSK9: A emerging participant in heart failure. BioMed Pharmacother. (2023) 158:114106. doi: 10.1016/j.biopha.2022.114106, PMID: 36535197

[17] Montecucco F Carbone F Liberale L Sahebkar A . Challenges in reducing atherosclerotic inflammation in patients with familial hypercholesterolemia. Eur J Prev Cardiol. (2020) 27:2099–101. doi: 10.1177/2047487319862907, PMID: 31288540

[18] Wang Y Fang X Liu J Lv X Lu K Lu Y . PCSK9 in T-cell function and the immune response. biomark Res. (2024) 12:163. doi: 10.1186/s40364-024-00712-8, PMID: 39736777 PMC11687167

[19] Galli SJ Gaudenzio N Tsai M . Mast cells in inflammation and disease: recent progress and ongoing concerns. Annu Rev Immunol. (2020) 38:49–77. doi: 10.1146/annurev-immunol-071719-094903, PMID: 32340580

[20] Choi YJ Yoo JS Jung K Rice L Kim D Zlojutro V . Lung-specific MCEMP1 functions as an adaptor for KIT to promote SCF-mediated mast cell proliferation. Nat Commun. (2023) 14:2045. doi: 10.1038/s41467-023-37873-3, PMID: 37041174 PMC10090139

[21] Li K Wang SW Li Y Martin RE Li L Lu M . Identification and expression of a new type II transmembrane protein in human mast cells. Genomics. (2005) 86:68–75. doi: 10.1016/j.ygeno.2005.03.006, PMID: 15953541

[22] Perrot CY Karampitsakos T Unterman A Adams T Marlin K Arsenault A . Mast-cell expressed membrane protein-1 is expressed in classical monocytes and alveolar macrophages in idiopathic pulmonary fibrosis and regulates cell chemotaxis, adhesion, and migration in a TGFbeta-dependent manner. Am J Physiol Cell Physiol. (2024) 326:C964–77. doi: 10.1152/ajpcell.00563.2023, PMID: 38189137 PMC11193480

